# High photocatalytic activity of V-doped SrTiO_3_ porous nanofibers produced from a combined electrospinning and thermal diffusion process

**DOI:** 10.3762/bjnano.6.132

**Published:** 2015-06-09

**Authors:** Panpan Jing, Wei Lan, Qing Su, Erqing Xie

**Affiliations:** 1 School of Physical Science and Technology, Lanzhou University, Lanzhou 730000, People's Republic of China,; 2 Key Laboratory for Magnetism and Magnetic Materials of Ministry of Education, Lanzhou University, Lanzhou 730000, People’s Republic of China

**Keywords:** electrospinning, photocatalysis, porous nanofibers, SrTiO_3_, thermal diffusion, vanadium-ion doping

## Abstract

In this letter, we report a novel V-doped SrTiO_3_ photocatalyst synthesized via electrospinning followed by a thermal diffusion process at low temperature. The morphological and crystalline structural investigations reveal not only that the V-doped SrTiO_3_ photocatalyst possesses a uniform, porous, fibrous structure, but also that some V^5+^ ions are introduced into the SrTiO_3_ lattice. The photocatalytic capability of V-doped SrTiO_3_ porous nanofibers was evaluated through photodegrading methyl orange (MO) in aqueous solution under artificial UV–vis light. The results indicated that V-doped SrTiO_3_ porous nanofibers have excellent catalytic efficiency. Furthermore, the excellent catalytic activity was maintained even after five cycle tests, indicating that they have outstanding photocatalytic endurance. It is suggested that the excellent photocatalytic performance of doped SrTiO_3_ nanofibers is possibly attributed to the V^5+^ ion doping increasing the light utilization as well as to the outstanding porous features, the excellent component and structure stability.

## Introduction

Along with the rapid advancement of heavy industrialization, environmental pollution and excessive nonrenewable energy consumption have emerged as global issues [1–2]. The most common issue is that many rivers and lakes are polluted by wastewater directly discharged from upstream factories, which seriously threatens aquatic organisms and human life. To purify the wastewater, several conventional treatment operations such as screening, sedimentation, and adsorption have been utilized [3]. Nevertheless, such operations cannot remove persistent and toxic soluble contaminants, such as organic dyes, chemical fertilizers and phenol [4]. Fortunately, a new and eco-friendly photocatalysis technique has drawn much attention. Photocatalysts are capable of accelerating the oxidation and mineralization of such organic substances with a fast removal rate [5–6] by producing strongly reactive and nonselective species, such as hydroxyl radicals (·OH) and superoxide anions (O_2_^−^). Since Fujishima and Honda first reported photo-electrochemical water splitting using a TiO_2_ electrode [7], many studies have been carried out on photocatalytic pollutant removal and electronic structures of semiconductors containing d^0^ metal ions [8–11], such as Ti^4+^, Zr^4+^, Ta^5+^, Nb^5+^ and V^5+^, as well as the development of new photocatalysts.

Strontium titanate (SrTiO_3_), an important multifunctional semiconductor, has been applied in photocatalysis technology for water splitting and organic contaminant degradation [12–13]. Although a promising photocatalytic candidate, the catalytic activity of SrTiO_3_ is still heavily influenced by its considerably large band gap of ≈3.25 eV and high dielectric permittivity [14]. The calculated band structure of SrTiO_3_ shows that the top of the valence band (VB) and the bottom of the unoccupied conduction band (CB) are composed of the O 2p and Ti 3d-t_2g_ states, respectively [15]. Due to the small contribution of Sr to the orbital characteristics of the conduction band, the energy difference between the O 2p and Ti 3d states mainly causes the band structure and insulation characteristic of SrTiO_3_. Previous works showed that doping with 3d (V, Fe, Ni) and 4f (Nd, Sm, Er) ions can significantly decrease the band gap through the hybridization of the Ti-3d and dopant-d states [16–17]. Additionally, the doped SrTiO_3_ also has an improved conductivity. Several groups have reported the excellent photocatalytic properties of Fe-doped SrTiO_3_, Nd-doped SrTiO_3_ and Ni/La co-doped SrTiO_3_ [18–20]. These new photocatalysts enable a good response to light or overcome light corrosion caused by the excessive accumulation of photogenerated carriers due to poor conductivity. However, there are few photocatalytic studies for V-doped SrTiO_3_ nanomaterials.

Thermal diffusion has been extensively applied to ion doping because it can effectively avoid the formation of a second phase in the host matrix [21–22]. Herein, pure SrTiO_3_ porous nanofibers are prepared by electrospinning, which is a more versatile, economic and simple approach to the preparation of 1D organic or inorganic nanomaterials [23–24]. This is followed by doping of V ions by a low temperature, thermal diffusion process. The photodegradation measurement indicates that V-doped, SrTiO_3_ porous nanofibers show an enhanced photocatalytic activity with excellent endurance.

## Results and Discussion

**Figure 1:**
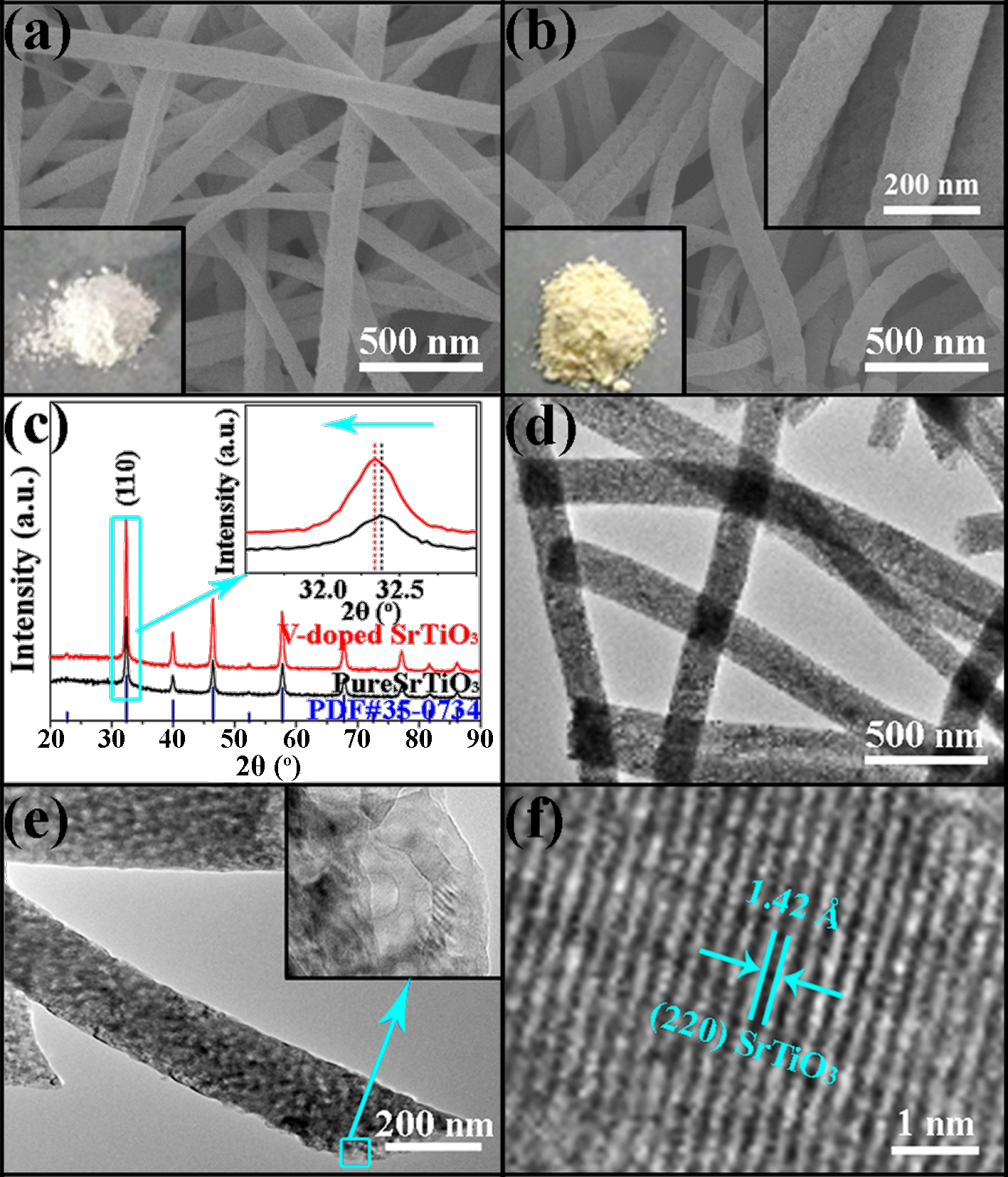
SEM images of (a) pure SrTiO_3_ and (b) V-doped SrTiO_3_ porous nanofibers, (c) XRD patterns, (d,e) TEM images and (f) HRTEM image of V-doped SrTiO_3_ porous nanofibers. The inset in (a) is a digital picture of pure SrTiO_3_ powders; the left lower and right upper insets in (b) are a digital photo and a high-magnification SEM image of V-doped SrTiO_3_ powders.

The morphology and microstructure are very important for the development of an excellent photocatalyst. In Figure 1a, the pure SrTiO_3_ nanofibers appear to be tens of micrometers in length, with a porous surface and uniform diameter distribution. The pore size and diameter distributions were measured to be about 10–32 nm and 90–240 nm, respectively. Such a long fibrous and porous structure is beneficial to electron transfer, dye molecular absorption and the light utilization efficiency for a photocatalyst. In Figure 1b, the morphological properties of doped samples appear similar to that of pure SrTiO_3_ nanofibers. From the insets in Figure 1a,b, the resulting doped SrTiO_3_ powders appear to be light yellow after immersing the white pure SrTiO_3_ powders in a NH_4_VO_3_ solution and further heat treatment in air. Figure 1c shows the typical XRD patterns of two samples. All the diffraction peaks could be indexed to the standard perovskite phase of SrTiO_3_ (JCPDS No. 35-0734) [25] without any indication of other impurity phases. Both samples show a polycrystalline structure, while the peaks of the V-doped SrTiO_3_ nanofibers are stronger and integrally shifted to shorter angles compared to that of pure SrTiO_3_ nanofibers. The inset of Figure 1c shows the relative position of the strongest peaks for the two samples. The peaks of the V-doped SrTiO_3_ nanofibers are stronger than that of pure SrTiO_3_ nanofibers, meaning the former has better crystallinity. Using the Debye–Scherrer equation [26], the average grain size of pure and V-doped SrTiO_3_ porous nanofibers were calculated to be about 20.4 and 21.8 nm, respectively. As illustrated by TEM images shown in Figure 1d,e, there are many uniformly distributed pores on the whole surface of the V-doped SrTiO_3_ nanofibers, which is consistent with the results from the SEM image. Moreover, Figure 1f displays a HRTEM image of V-doped SrTiO_3_. The average fringe spacing was measured to be about 1.42 Å which is larger than the 1.38 Å of the (220) plane of standard SrTiO_3_. Correlating these XRD results, it could be deduced that a few V ions were likely incorporated into the SrTiO_3_ lattice which then induced the expansion of the SrTiO_3_ lattice.

**Figure 2:**
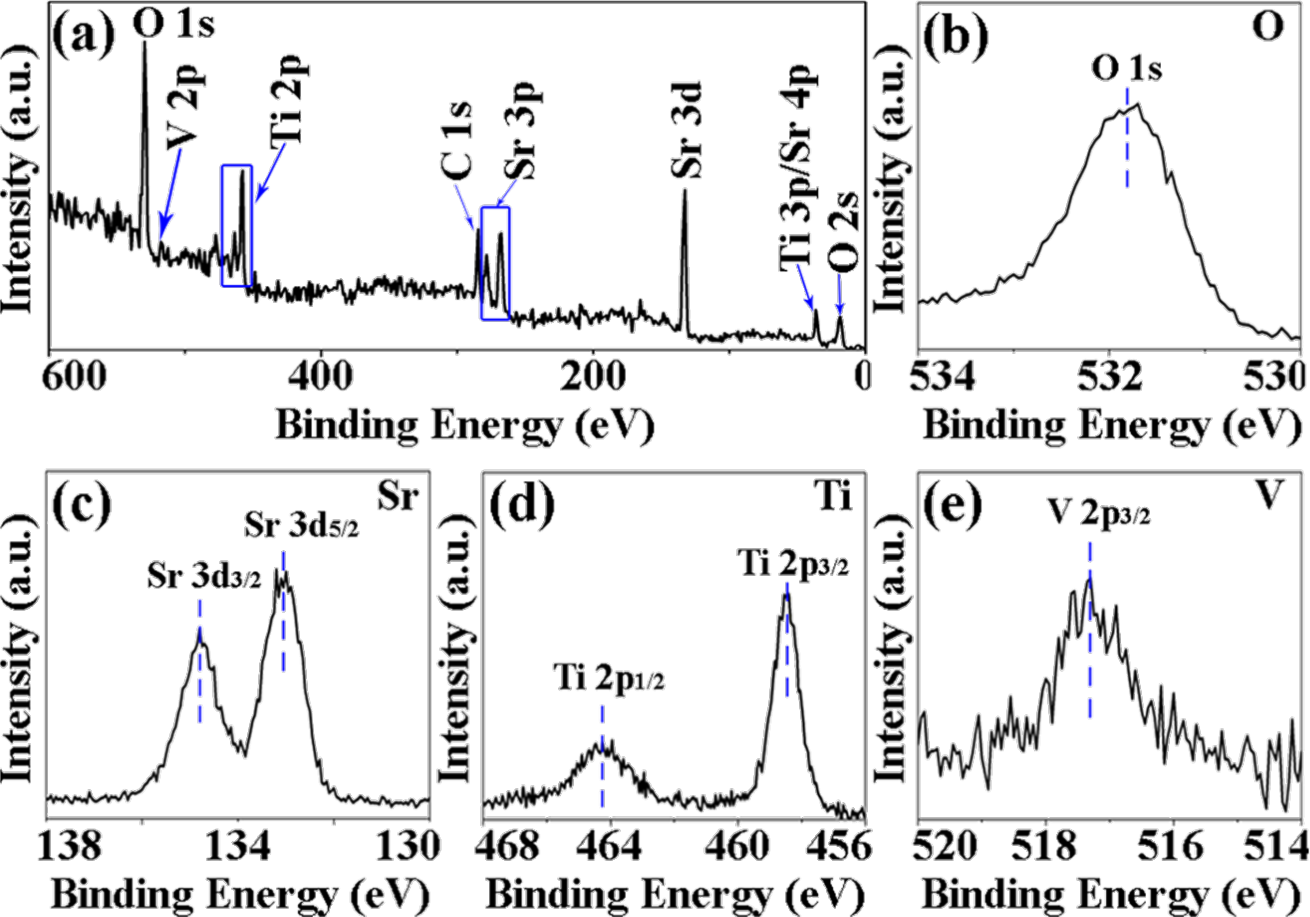
(a) Full XPS spectrum of the V-doped SrTiO_3_ porous nanofibers, (b–e) high-resolution XPS spectra of O, Sr, Ti and V, respectively.

**Figure 3:**
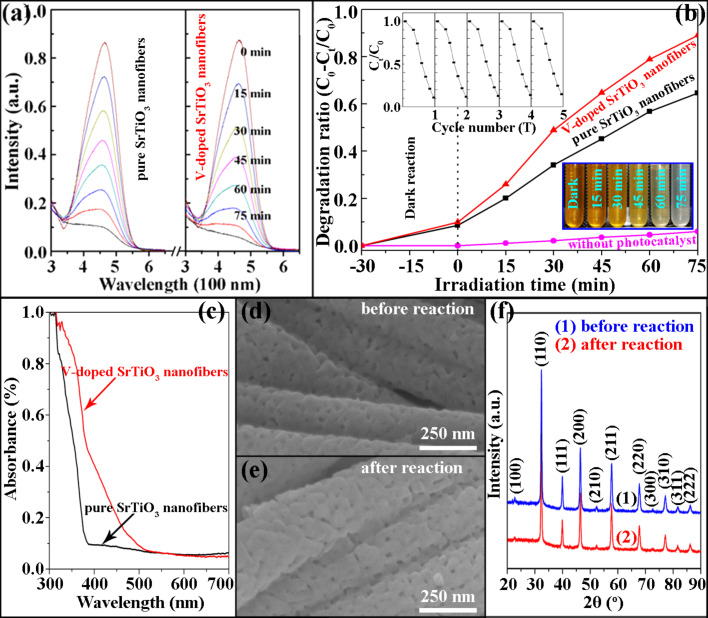
(a) UV–vis absorbance spectra of a MO solution monitored during the catalytic reaction. (b) Degradation ratio (*C*_0_−*C_t_*/*C*_0_) vs irradiation time (*t*) of MO. The insets in (b) are cycling tests of V-doped SrTiO_3_ porous nanofibers (upper left) and digital pictures of the MO solutions at different times (lower right). (c) UV–vis spectra of the two catalysts. (d,e) SEM images and (f) XRD patterns of V-doped SrTiO_3_ porous nanofibers before and after catalytic reaction.

XPS analysis was performed to determine the elemental composition and chemical states of V-doped SrTiO_3_ porous nanofibers. All XPS data were corrected by reference to the C 1s peak at about 284.8 eV. As shown in Figure 2a, the complete XPS spectrum reveals that the elements Sr, Ti, V and O coexist in V-doped SrTiO_3_ porous nanofibers. The atomic ratio of V/Sr/Ti is estimated to be about 1.9:48.6:49.5, meaning that the V ion concentration doped into SrTiO_3_ is 3.5 atom %. The high-resolution XPS spectra of O 1s (Figure 2b), Sr 3d (Figure 3c), and Ti 2p (Figure 2d) are similar to the earlier reports of SrTiO_3_ [27–28]. The peak positioned at about 517.3 eV shown in Figure 2e is labeled as V^5+^ [29]. It has been reported that SrTiO_3_ presents a perfect cubic perovskite structure above 105 K, where Sr^2+^ ions are at the corner of the cube and a Ti^4+^ ion occupies the centrosymmetric position surrounded by six O^2−^ anions, forming a TiO_6_ octahedron [30]. In other words, the crystalline structure of SrTiO_3_ is a framework of O^2−^ anions. When a sample is fabricated above room temperature in air, generally, the oxygen vacancy is one of the most important defects that can be easily introduced [31]. The crystal cell is then distorted and the cell volume or lattice constant is reduced. However, if a few Ti^4+^ ions are substituted by V^5+^ ions, some oxygen vacancies will be filled by oxygen, owing to balance between the positive and negative charges. The lattice of the V-doped SrTiO_3_ increases and is larger than that of pure SrTiO_3_, which is similar with the result observed from XRD and HRTEM. Hence, it is concluded that V-doped SrTiO_3_ nanofibers were successfully synthesized.

The photocatalytic activity of pure and V-doped SrTiO_3_ porous nanofibers were evaluated by the decomposition of methyl orange (MO) in aqueous solution under UV–vis light irradiation. In Figure 3a, the characteristic absorption peak of MO at approx. 464 nm is given which shows a progressive decrease with increasing irradiation time in the presence of pure and V-doped SrTiO_3_ porous nanofibers. No new absorption peaks were produced during the irradiation, which means that the MO is completely decomposed. Figure 3b displays the degradation ratio of MO with irradiation time (*C*_0_: initial concentration of MO, *C*_0_−*C_t_*: degraded concentration of MO at time *t*). This clearly demonstrates that only about 6% of the MO is degraded without any photocatalyst after irradiation for 75 min. In the case of the pure and V-doped SrTiO_3_ porous nanofibers, in contrast, the degraded MO is about 60% and >90%, respectively. Therefore, the self-photolysis of MO can be neglected. Furthermore, compared with pure SrTiO_3_ nanofibers, one can observe that porous V-doped SrTiO_3_ nanofibers exhibit an enhanced catalytic rate. Notably, the decrease of the MO concentration during the dark reaction could indicate that both of the two photocatalysts absorb the MO fluorescence well. Figure 3c presents the UV–vis spectra of pure and V-doped SrTiO_3_ photocatalyst, revealing that pure SrTiO_3_ responds to UV light with absorption edge at about 388 nm but V-doped SrTiO_3_ nanofibers have a shifted absorption edge towards visible light. This means that V^5+^ ion doping improves the impurity level and narrows the band gap of SrTiO_3_, leading to the enhanced light absorption. Additionally, based on earlier research regarding 3d ion doping for SrTiO_3_, V^5+^ doping may also improve the conductivity of SrTiO_3_. Hence, the higher photocatalytic activity of V-doped SrTiO_3_ nanofibers compared to pure SrTiO_3_ nanofibers can be attributed to the V^5+^ ion doping. Stability and reusability are also very important for photocatalysts. As shown in the upper left inset in Figure 3b, the catalytic efficiency of V-doped SrTiO_3_ nanofibers is barely changed after five catalytic reaction recycles, indicating the high photocatalytic endurance of the V-doped SrTiO_3_ nanofibers. This result was supported by the structure and component durability revealed from the SEM images (Figure 3d,e) and XRD patterns (Figure 3f) characterized before and after the photocatalytic reaction. Accordingly, the V-doped SrTiO_3_ porous nanofibers could be a promising candidate for the clean-up of industrial waste water.

In this work, it is suggested that there are three main contributors to the remarkable photocatalytic performance of V-doped SrTiO_3_ porous nanofibers. Firstly, the porous structure results in more reaction sites to promote the incident light utilization and reaction sites between adsorbed dye molecules and oxidizing ions (O^2−^, ·OH, etc.). Secondly, V^5+^ ion doping provides an impurity level in the energy gap of SrTiO_3_ to improve the light response and conductivity. Thirdly, the V-doped SrTiO_3_ porous nanofibers also have considerable structure and phase stability. For a better understanding of the photocatalytic system of doped SrTiO_3_, more detailed investigations regarding V and other 3d ions co-doped into SrTiO_3_ are being carried out.

## Conclusion

In conclusion, V-doped SrTiO_3_ porous nanofibers were successfully synthesized via electrospinning followed by a thermal diffusion process. They were subsequently characterized by FESEM, TEM, XRD, XPS and UV–vis spectra in detail. During the photodisintegration of methyl orange (MO) under UV–vis light irradiation, the V-doped SrTiO_3_ porous nanofibers exhibited excellent photocatalytic activity. Furthermore, the cycling testing also confirms its outstanding catalytic endurance. The synergistic effect of V ion doping and the porous character is applied to understanding the excellent photocatalytic capability of V-doped SrTiO_3_ porous nanofibers. We believe that this work will benefit the fundamental research of 3d-ion-doped SrTiO_3_ as well as its photocatalysis applications.

## Experimental

### Synthesis of V-doped SrTiO_3_ porous nanofibers

All chemical reagents were analytically pure and used without further purification. A typical preparation procedure as follows. Firstly, pure SrTiO_3_ nanofibers were prepared via electrospinning followed by heat treatment. 0.25 g of poly(vinylpyrrolidone) (PVP, *M*_w_ = 1,300,000) and 0.34 g of Ti(C_4_H_9_O)_4_ were completely dissolved in a mixed solvent comprised of 0.6 g *N,N*-dimethylformamide (DMF), 1.0 g CH_3_COOH and 1.0 g C_2_H_5_OH under vigorous stirring. Meanwhile, a Sr(NO_3_)_2_ solution was also prepared by dissolving 0.147 g pf SrCO_3_ in 1.3 g of dilute HNO_3_ (15%). About 6 h later, a homogenous spinning emulsion with stoichiometric Sr^2+^ and Ti^4+^ was obtained by sufficiently mixing the above solutions. This was then transferred to a glass syringe with a stainless steel needle (inner diameter ≈0.4 mm) for electrospinning. The distance and voltage from the tip of needle to the collector were set at 20 cm and 17 kV, respectively. The feeding rate of the emulsion was set at 0.5 mL/h. After electrospinning, the as-spun nanofibers were annealed at 600 °C for 2 h with a heating rate of 2 °C/min. Then a portion of the resulting pure SrTiO_3_ nanofibers were immersed in a 20 g (1 wt %) NH_4_VO_3_ solution at 60 °C for overnight. Finally, the precipitates were separated, dried and annealed in air at 300 °C for another 2 h, and V-doped SrTiO_3_ porous nanofibers were then obtained.

### Characterization and photocatalytic evaluation of V-doped SrTiO_3_ porous nanofibers

The surface morphology, crystal structure, chemical composition and optical properties of pure and V-doped SrTiO_3_ porous nanofibers were characterized using field emission scanning electron microscopy (FESEM, Hitachi S-4800) and transmission electron microscopy (TEM, Tecnai^TM^ G^2^F30, FEI), X-ray diffraction (XRD, Cu Kα, λ = 1.5406 Å), high-resolution transmission electron microscopy (HRTEM) and X-ray photoelectron spectroscopy (XPS, Kratos AXIS Ultra^DLD^, monochrome Al target) and UV–vis spectrophotometry (U-3600). The degradation of methyl orange (MO) in aqueous solution was used to evaluate the catalytic activity of the as-prepared photocatalysts. In a typical photocatalytic experiment, 30 mg of photocatalyst powder and 40 mL of MO solution (10 mg/L) were loaded together in a beaker (100 mL). The mixed solution was first stirred in the dark for 45 min to achieve an adsorption–desorption equilibrium between the dye and catalysts. The beaker was then exposed to a 175 W mercury lamp. During irradiation, an approximately 4.5 g suspension was sampled at regular 15 min intervals. The change in the MO concentration was monitored by recording the absorption peak maximum at about 464 nm of the MO via UV–vis spectrophotometry. After each catalytic reaction, the used photocatalysts powders were recycled by centrifuging at 3000 rpm and drying. They were then used for the next cycle. In this work, we performed five cycling tests to verify the catalytic endurance of V-doped SrTiO_3_ porous nanofibers.

## References

[1] Wang M, Ioccozia J, Sun L, Lin C, Lin Z (2014). Energy Environ Sci.

[2] Alic J, Sarewitz D, Weiss C, Bonvillian W (2010). Nature.

[3] Sonune A, Ghate R (2004). Desalination.

[4] Murgolo S, Petronella F, Ciannarella R, Comparelli R, Agostiano A, Curri M L, Mascolo G (2015). Catal Today.

[5] Yu B Y, Kwak S-Y (2012). J Mater Chem.

[6] Senapati S, Srivastava S K, Singh S B (2012). Nanoscale.

[7] Fujshima A, Honda K (1972). Nature.

[8] Niishiro R, Kato H, Kudo A (2005). Phys Chem Chem Phys.

[9] Mishima T, Matsuda M, Miyake M (2007). Appl Catal, A.

[10] Oshikiri M, Boero M, Ye J, Zou Z, Kido G (2002). J Chem Phys.

[11] Li G-L, Yin Z (2011). Phys Chem Chem Phys.

[12] Niishiro R, Tanaka S, Kudo A (2014). Appl Catal, B: Environ.

[13] Puangpetch T, Sreethawong T, Yoshikawa S, Chavadej S (2008). J Mol Catal A: Chem.

[14] Reunchan P, Ouyang S, Umezawa N, Xu H, Zhang Y, Ye J (2013). J Mater Chem A.

[15] van Benthem K, Elsässer C, French R H (2001). J Appl Phys.

[16] Maletic S, Maletic D, Petronijevic I, Dojcilovic J, Popovic D M (2014). Chin Phys B.

[17] Baniecki J D, Ishii M, Aso H, Kurihara K, Ricinschi D (2013). J Appl Phys.

[18] Xie T-H, Sun X, Lin J (2008). J Phys Chem C.

[19] Zheng J-Q, Zhu Y-J, Xu J-S, Lu B-Q, Qi C, Chen F, Wu J (2013). Mater Lett.

[20] Jia A, Liang X, Su Z, Zhu T, Liu S (2010). J Hazard Mater.

[21] Zhang Z, Chen Q, Lee H D, Xue Y Y, Sun Y Y, Chen H, Chen F, Chu W-K (2006). J Appl Phys.

[22] Phan T L, Vincent R, Cherns D, Nghia N X, Ursaki V V (2008). Nanotechnology.

[23] Luo C J, Stoyanov S D, Stride E, Pelan E, Edirisinghe M (2012). Chem Soc Rev.

[24] Yang G, Yan W, Wang J, Zhang Q, Yang H (2014). J Sol-Gel Sci Technol.

[25] Kimijima T, Kanie K, Nakaya M, Muramatsu A (2014). Appl Catal, B: Environ.

[26] Holzwarth U, Gibson N (2011). Nat Nanotechnol.

[27] Yu H, Wang J, Yan S, Yu T, Zou Z (2014). J Photochem Photobiol, A.

[28] Marshall M S J, Newell D T, Payne D J, Egdell R G, Castell M R (2011). Phys Rev B.

[29] Zhao W, Zhong Q (2014). RSC Adv.

[30] da Silva L F, Avansi W, Andrés J, Ribeiro C, Moreira M L, Longo E, Mastelaro V R (2013). Phys Chem Chem Phys.

[31] Yang Q, Cao J X, Zhou Y C, Zhang Y, Ma Y, Lou X J (2013). Appl Phys Lett.

